# Zika virus knowledge and attitudes in Ecuador

**DOI:** 10.3934/publichealth.2018.1.49

**Published:** 2018-03-26

**Authors:** Sharon L. Casapulla, Gloria Aidoo-Frimpong, Tania B. Basta, Mario J. Grijalva

**Affiliations:** 1 Office of Rural and Underserved Programs, Ohio University Heritage College of Osteopathic Medicine, Athens, OH, United States; 2 Infectious and Tropical Disease Institute, Department of Biomedical Sciences, Heritage College of Osteopathic Medicine, Athens OH, United States; 3 Department of Social and Public Health, College of Health Sciences and Professions, Ohio University, Athens OH, United States; 4 Center for Research on Health in Latin America, School of Biological Sciences, Pontifical Catholic University of Ecuador, Quito, Ecuador

## Abstract

Since its discovery in 1947 in Uganda, ZIKV has spread to 61 countries with a total of 229,238 confirmed human cases worldwide. Specifically, Ecuador has recorded 3,058 confirmed cases and 7 confirmed cases of congenital syndrome associated with ZIKV. Using the Health Belief Model (HBM), this pilot study was conducted to assess Zika virus-related knowledge and attitudes among adults in Ecuador. The survey data were collected in public places in rural and urban areas of Ecuador in May 2016. Seven items measured ZIKV knowledge and 23 items measured attitudes toward ZIKV. A total of 181 Ecuadorians participated in this study. The average age of the sample was 33.4. With respect to ZIKV knowledge, the majority of the participants had heard of ZIKV (n = 162, 89.5%). More males reported first hearing of ZIKV on the internet (*p* = 0.02), more rural individuals reported knowing someone diagnosed with ZIKV (*p* = 0.02), more primary school educated individuals reported hearing about ZIKV first from their doctor/nurse (*p* = 0.03), and more high school graduates correctly identified that ZIKV could be transmitted from mother to child (*p* = 0.03). As for the HBM constructs, there was a statistically significant difference between gender and cues to action (*p* = 0.04), with males having a statistically significant lower mean on the cues to action items compared to females. There were also statistically significant differences between those categorized as having “adequate” knowledge compared to “low” knowledge on the benefits construct (*p* = 0.04) and the perceived severity construct (*p* = 0.03). There is a clear need for education about the transmission and prevention of ZIKV. High levels of self-efficacy for prevention behaviors for ZIKV combined with low perceived barriers in this community set the stage for effective educational interventions or health promotion campaigns that can ameliorate the knowledge deficits surrounding transmission and prevention.

## 1. Introduction

The Zika virus (ZIKV) was isolated for the first time in 1947 in the Zika forest in Uganda [1],[2]. On February 1, 2016, the World Health Organization (WHO) declared ZIKV a public health emergency of international concern [2]. In March 2017, the WHO reported that 84 countries have found evidence of mosquito-borne transmission of ZIKV and 61 countries have reported human ZIKV cases [3]. At the end of November 2017, the total number of confirmed human ZIKV cases worldwide was 229,238 with an incidence rate of 80.19 per 100,000 [4]. The Pan American Health Organization (PAHO) reported nearly 500,000 suspected ZIKV cases in Central American, South American and Caribbean countries [5]. Despite reports indicating that some countries in South America are reporting a decrease in incidence of ZIKV cases [5], the WHO recommends that vigilance remain high [3]. In Ecuador, recent reports show a decrease in suspected cases but increase in number of confirmed cases of ZIKV [6]. *Aedes* mosquitoes transmit the Zika virus. Onset of the disease is usually 2–7 days after the mosquito bite and is usually accompanied by rash, mild fever, conjunctivitis, and muscle pain. Only 25% of those infected will develop symptoms [7],[8]. The virus has been found in semen and can be sexually transmitted [9]. Since February 2015, 13 countries have reported person to person transmission [3]. Five countries in the Americas have reported sexually transmitted ZIKV cases [5]. ZIKV can be transmitted in utero to the unborn fetus but there are reports of ZIKV being found in breastmilk [10].

ZIKV is a public health issue because of the severe consequences of infection that can occur. Infection in pregnant women, specifically, is of major concern as it is linked to catastrophic fetal abnormalities including microcephaly, spontaneous abortion, and intrauterine growth restriction [7]. Research has shown that ZIKV infection during pregnancy can cause congenital brain abnormalities, including microcephaly, which can produce both mental and physical developmental disabilities [11]–[13]. ZIKV can also trigger Guillain-Barre syndrome (GBS), a neurological condition that causes the body's immune system to attack the nervous system, resulting in varying degrees of weakness, tingling, and/or paralysis [14]. Because of the complications, it is essential that appropriately designed interventions to communicate credible information about ZIKV are implemented, especially in Ecuador where there are active cases of transmission. Two cases of GBS have been reported in Ecuador [6].

Public health researchers recognize that risk reduction includes dealing with information gaps in one of the three pillars in the “Zika Triad” [15] along with reducing disease spread and recognizing vulnerabilities. The ZIKV triad consists of an external agent, a susceptible host, and an environment that brings the host and agent together. In this case, disease results from the interaction between the agent and the susceptible host in an environment that supports transmission of the agent from a source to that host [15]. Therefore, understanding how individuals think about their susceptibility to ZIKV, the consequences of getting ZIKV, as well as their beliefs about their ability to prevent transmission of the disease are critical to developing effective methods for reducing risk and ultimately, transmission rates.

In Ecuador, recent reports show a decrease in suspected cases but increase in number of confirmed cases of ZIKV [6]. To date, Ecuador (population 16,279,000) has reported 3,753 suspected and 3,058 confirmed autochthonous cases of ZIKV (incidence rate of 41.26/100,000) [4]. The highest ZIKV incidence rates were reported from the coastal provinces of Esmeraldas, Guayas, Manabi, and Santo Domingo de los Tsáchilas (incidence rate of 23.0–201.8/100,000) [6]. As of epidemiological week (EW) 32 of 2017, there were 912 confirmed cases in pregnant women, with the highest number of confirmed cases (329 cases) from Guayas Province, which is near the coast and was somewhat affected by the earthquake in April, 2016. Of the total cases in pregnant women, 176 were infected in the first trimester, 531 in the second and 205 in the third [6]. There have been 7 confirmed cases of congenital syndrome associated with ZIKV in Ecuador since 2015 [4]. No deaths have been reported in Ecuador related to ZIKV [6].

The health belief model (HBM) [16]–[18] is one of the most widely utilized value-expectancy theories in public health and has been applied to numerous health behaviors. The model posits that if a person holds expectancies about a particular disease or condition and the person believes there is a behavior(s) that can prevent the disease or condition, then the individual is more likely to perform the associated behavior(s). The HBM has 6 constructs, including perceived susceptibility (susceptibility to ZIKV), perceived severity (consequences of getting ZIKV), perceived barriers (barriers to preventing ZIKV), perceived benefits (benefits to performing behaviors to prevent ZIKV), cues to action (cues to prompt behavior change to prevent ZIKV) and self-efficacy (the confidence to prevent ZIKV).

Previous research has been conducted on knowledge and perceptions of Zika and ZIKV in several countries including Colombia [19], India [20], Nigeria [21], Qatar [22], as well as in the US [23]. The study in Colombia utilized a convenience sample (n = 269) of participants (healthcare workers and students) in a ZIKA symposium across four locations. Not surprisingly, the levels of knowledge of ZIKV were high [19]. The study in India assessed Zika knowledge among dental practitioners (n = 412) in a highly urbanized area. Less than half of the practitioners had high levels of knowledge of Zika. The researchers found differences in mean knowledge scores based on qualification of the participants, with postgraduates having more knowledge than graduates. Television was the primary source of information for most of the respondents [20]. A study of 377 reproductive-age women attending a Nigerian general outpatient clinic found that overall the women had high levels of awareness of Zika (including that it caused microcephaly) but low levels of knowledge of transmission, and in particular lacked knowledge of prevention of sexual transmission [21]. The study in Qatar also utilized a convenience sample of university students (n = 446). The authors concluded that the population had inadequate knowledge of Zika, and felt that even though few cases had been reported, health education about Zika is important in this population [22].

While the aforementioned studies represent some of the emerging studies assessing ZIKV knowledge and attitudes, to date few published studies have assessed the attitudes toward ZIKV using the HBM and none has done so in Latin America. Therefore, the purpose of this study was to assess (1) ZIKV knowledge and (2) attitudes toward ZIKV based on the constructs of the HBM. The results of this study will be used to assist in intervention design and messaging in Ecuador.

## 2. Materials and Methods

### 2.1. Study setting and study sites

This study utilized a cross-sectional design in which self-report survey data were collected in multiple community settings in Ecuador. This study was a part of a larger study that was approved by the Institutional Review Board at Ohio University (IRB protocol #16x117). Survey data were collected at parks, workplaces, markets, hospital and public health clinics in rural and urban settings in Ecuador. Data were collected in May 2016 from small cities and rural areas in the Amazon and Andes region of Ecuador and spanned from southern Ecuador near the Peruvian border to Northern Ecuador. These data were collected as part of a 15 day study abroad trip to Ecuador, during which 6 localities were visited. These locations were chosen for the study abroad experience and not able to be altered for data collection. Since data collection was not the only purpose of the study abroad, and there were very few days in each location, only a few hours of data collection were possible at each site. Within each of the 6 locations, as previously mentioned, data were collected by multiple groups at multiple settings (For example, a group of 3–4 would go to the market, while others would go to the park or health clinic). Target recruitment was not pre-determined in each location. Data were collected time permitting at each site.

This study was part of a larger study that collected self-report quantitative data about HIV knowledge, HIV stigma, attitudes toward safer sex, attitudes toward teen pregnancy, and ZIKV knowledge and attitudes. As such, the full study instrument was 13 pages long and had 136 items. Initially, assessing ZIKV knowledge and attitudes was not a part of the original study, but the items were added due to the timely nature of the epidemic. For the larger study, all the instruments, except for the attitudes toward teen pregnancy and ZIKV knowledge and attitudes, were found to be valid and reliable in English speaking populations. The HIV knowledge had also been validated in Spanish speaking individuals in the United States. To the knowledge of these authors, this was the first time any of these scales had been used in Ecuador. The teen pregnancy and ZIKV items were developed by the author and reviewed for face validity by content experts. It took participants 20–30+ minutes (depending on literacy levels) to complete the questionnaire, and unfortunately, the ZIKV items were last. As a result, twenty-three participants of the larger study did not complete the entire questionnaire; leaving the ZIKV items blank.

### 2.2. Participant recruitment

The research team consisted of 10 native English-speaking university students and 4 bilingual individuals (3 Ecuadorian and 1 American). Teams of 4, including a bilingual individual, visited various aforementioned locations to distribute the surveys. To be eligible to participate, individuals had to meet the following inclusion criteria: (1) 18 years of old or older, (2) live in Ecuador, and (3) speak fluent Spanish. Participants were approached in public spaces and were invited to read the informed consent form in Spanish or have it read to them. If they consented to participate and signed the informed consent form, they were given the survey in Spanish to complete on their own, or with assistance if needed.

Number and percentage of refusals were not tracked, but it was clear that in the rural indigenous areas, the refusal rates were higher. In all locations, one of the authors and her bilingual Ecuadorian assistant had visited with the local health officials and/or practitioners in summers 2014 and 2015 to conduct an informal needs assessment. It was during these trips that the locals made it clear that sexual health was a priority due to the high teen pregnancy rate. As a result, in summer 2016, the authors designed the larger study to assess sexual health related topics. Since the authors had visited these locations 2 times prior and were known and respected in the local communities. Even though this was the case, the research team had the most trouble collecting data from individuals in Saraguro, despite having met with the Mayor of Saraguro the two previous summers and getting his approval to collect data on these sensitive issues.

Most of the participants completed the surveys in about 20–30 minutes and were given information about ZIKV and HIV in exchange for their time. The ZIKV information that we distributed was a one-page pictogram developed by the CDC in Spanish and the HIV prevention information was distributed in a small pamphlet called “Me Quede Frio”, which was developed and distributed by an Ecuadorian non-profit, Fundacion Ecuatoriana Equidad. We also distributed condoms to interested individuals. Condoms that were not distributed were donated to public health clinic in a small rural community outside of Tena.

### 2.3. Measures

The measures in this study assessed knowledge and attitudes related to ZIKV. There were 7 items that measured ZIKV knowledge and there were 23 items that measured attitudes toward ZIKV using the Health Belief Model (HBM). These knowledge and HBM attitude items were developed by one of the authors based on transmission information that was available by CDC in early 2016, because at the time this study was designed and submitted for IRB review, the WHO survey instrument was not available [24]. The knowledge items were reviewed for face validity by an expert in infectious diseases and the attitude items were similarly reviewed by experts in health behavior. The HBM knowledge items were either dichotomous (yes/no or true/false) or multiple choice and multiple response (choose all that apply). The HBM items were scored on a Likert scale from 1 (strongly disagree) to 5 (strongly agree). The total possible scores on the scale ranged from 23 to 115. The HBM items measured each of the six HBM constructs, including perceived susceptibility to ZIKV (range possible 2–10), perceived severity of acquiring ZIKV (range possible 3–15), perceived benefits of prevention activities (range 5–25), perceived barriers to prevention activities (range possible 5–25), self-efficacy to prevent ZIKV (range possible 5–25), and cues to action to prompt prevention activities range 3–15). (See Table 1 for wording of questions). Demographic information was also collected for each participant, including age, gender, marital status, educational background and pregnancy status (if female).

**Table 1.** ZIKV Health Belief Model constructs (HBM) among Ecuadorian population (N = 162).

Construct items	N*	%
*Perceived Susceptibility*		
I am at risk for getting ZIKV	77	47.5
I am at greater risk of getting ZIKV than other people	40	24.7
*Perceived Severity*		
ZIKV may cause serious health problems	126	77.8
ZIKV complications are dangerous	120	74.1
If I contracted the ZIKV, it could spread to other family members	92	56.8
*Perceived Benefits to Preventing Zika Virus*		
Preventing mosquito bites can prevent ZIKV infection	108	66.7
Wearing bug spray can prevent ZIKV infection	118	72.8
Wearing long shirts and pants can prevent ZIKV infection	103	63.6
Wearing condoms can prevent ZIKV transmission to another person	45	27.8
*Perceived Barriers to Preventing Zika Virus*		
I am generally opposed to using condoms	30	18.5
Bug spray is expensive	37	22.8
Bug spray is easy to obtain in my town	119	73.5
Condoms are expensive	29	17.9
Condoms are easy to obtain in my town	116	71.6
Wearing long sleeves and pants is easy for me to do	91	56.2
*Cues to Action*		
The media impacts my decision to take action to prevent getting ZIKV	111	68.5
My doctor/nurse impacts my decision to take action to prevent ZIKV	91	56.2
My friends/family impact my decision to take action to prevent ZIKV	92	56.8
*Self-Efficacy*		
I am confident that I can prevent getting ZIKV	104	64.2
I am confident I can wear bug spray	122	75.3
I am confident I can wear long sleeves/pants	126	77.8
I am confident I can wear condoms	104	64.2
I am confident I can avoid pregnancy	110	67.9

*Equals *Agree* and *Strongly Agree* combined.

Note: Wearing long clothing can prevent Zika X^2^(1, N=140) 6.72, p = 0.01 – rural urban.

T-test results by Knowledge Level and Individual Health Belief Items indicate *If I get ZIKV, it could spread to others in my family* (t(149) = −2.34, *p* = 0.02) and *Using insect repellent could prevent the spread of Zika* (t(152) = 2.85, *p* = 0.00) were statistically significant.

**Table2.** T-test results by Health Belief Construct.

Construct	Gender	Rural/Urban	Education	Vaccine Exists	Knowledge Level
Perceived Susceptibility	t(152) = 1.13, *p* = 0.225	t(152) = −1.15, *p* = 0.25	t(130) = 0.45, *p* = 0.66	t(152) = 0.79, *p* = 0.64	t(151) = −0.37, *p* = 0.97
Perceived Severity	t(150) = −1.73, *p* = 0.09	t(150) = 0.08, *p* = 0.93	t(129) = −0.78, *p* = 0.44	t(150) = 1.34, *p* = 0.18	t(149) = −2.17, *p* = 0.03*
Perceived Benefits	t(147) = −0.37, *p* = 0.71	t(147) = 0.05, *p* = 0.96	t(1287) = 1.19, *p* = 0.24	t(147) = 0.35, *p* = 0.73	t(146) = 2.12, *p* = 0.04*
Perceived Barriers	t(140) = −0.36, *p* = 0.72	t(140) = 1.26, *p* = 0.21	t(121) = 0.04, *p* = 0.97	t(140) = 0.53, *p* = 0.60	t(139) = .17, *p* = 0.87
Cues to Action	t(151) = −1.98, *p* = 0.04*	t(151) = 1.29, *p* = 0.20	t(129) = 0.48, *p* = 0.63	t(151) = 0.36, *p* = 0.72	t(150) = −0.12, *p* = 0.91
Self-Efficacy	t(144) = −0.33, *p* = 0.74	t(144) = −0.69, *p* = 0.49	t(122) = 0.27, *p* = 0.79	t(144) = −0.19, *p* = 0.86	t(143) = 0.05, *p* = 0.96

### 2.4. Data analysis

Descriptive statistics were used to describe the demographic data, knowledge and attitudinal data. Marital status was dichotomized to “partnered” or “unpartnered” and educational background was dichotomized to “high school graduate” or “not a high school graduate.” Paired t-tests were run to assess the differences in the HBM items by educational status, gender, and location of residence (rural vs. urban). Rural was designated for any location with less than 50,000 residents and urban for greater than 50,000 [25]. Separate cross-tab analyses were conducted to determine if a relationship existed between gender, educational level, rural/urban and ZIKV knowledge items. The chi-square test statistics and associated *p*-values were reported for all cross-tabs with 5 or more responses per cell and Fisher's Exact test *p*-values were reported for analyses with fewer than 5 per cell.

A ZIKV knowledge scale was developed using 4 of the 7 knowledge items. Two of the 4 items had multiple responses (choose all that apply) so the responses were transformed into unique variables with dichotomous (yes no) responses. For example, one item asked, “How is Zika transmitted?” and it had 7 possible responses (mosquito bites, sexual contact, mother to child, blood transfusion, urine or feces, casual contact, and coughing/sneezing). Each item checked was calculated as a “yes” dichotomous response and those left blank as “no.” The possible knowledge scores ranged from a low of 0 to a high of 13. Participants were classified as “adequate knowledge” if their score was 1 standard deviation above the mean or higher. All others were classified as “poor knowledge.” We only used 1 standard deviation so there would be enough cases for statistical analyses.

Separate independent sample t-tests were conducted with gender, rural/urban, educational level, knowledge level, and belief vaccine exists as grouping variables and the HBM constructs as the continuous dependent variables. If one of the HBM constructs had statistically significant differences between grouping variables, then additional t-tests were computed for the individual items that made up the HBM constructs. All analyses were conducted using SPSS Version 23.0 and 24.0 [26].

## 3. Results

### 3.1. Participant Characteristics

A total of 181 Ecuadorians agreed to participate in this study. The average age of the sample was 33.4 (SD ± 10.9) with a range from 18 to 65 years of age. Over half the sample lived in urban (n = 98) locations (population greater than 50,000), identified as female (n = 96, 53%) were married or partnered (n = 110, 60.8%), and had at least a high school education (n = 116, 64.1%). Of the 96 women in this study, only 3 were pregnant at the time of the study (See Table 3).

**Table 3.** Characteristics of study sample (N=181).

Characteristics	N	%
*Average age*	33.4 (SD ± 10.90)	
Range 18–65		
*Gender*		
Female	96	53.0
Male	85	47.0
Pregnant	3	
*Relationship*		
Married	110	60.8
Single	70	39.2
*Educational Level*		
Primary school only	41	22.7
Higher education	69	38.1
*Location*		
Saraguro	43	24.0
Loja	44	24.0
Tena	30	17.0
Carimanga	30	16.0
Otavalo	14	11.0
Mach	20	8.0

### 3.2. Knowledge of ZIKV

With respect to ZIKV knowledge, most of the participants had heard of ZIKV (n = 162, 89.5%) and of those who had, more than half (n = 87, 53.7%) first heard about ZIKV from the television. The majority knew that ZIKV was transmitted via mosquito bites (n = 151, 93.2%), but only 8.6% (n = 14) and 17.3% (n = 28) knew that it was transmitted sexually and from mother to child, respectively. While over half the sample (n = 102, 62.9%) knew that preventing a mosquito bite could prevent ZIKV transmission, only 9.8% (n = 16) knew that wearing condoms could prevent transmission. Notably, 50.0% (n = 81) erroneously believed that a vaccine was available to prevent ZIKV and only 21.6% (n = 35) of the participants, who knew that ZIKV was transmitted via mosquitoes, knew that the mosquitoes were day biters. Nearly 85% (n = 135) knew that there were confirmed cases of ZIKV in Ecuador and 14.2% (n = 23) knew someone who had been diagnosed with ZIKV (See Table 4). None of the analyses revealed differences for gender, educational level, and rural/urban and knowledge items.

There were differences by grouping variables (See Table 4). There were more males who reported hearing of ZIKV on the internet first compared to women, (*p* = 0.02), there were more rural individuals who reported knowing someone diagnosed with ZIKV (*p* = 0.02) compared to their urban counterparts, there were more primary school educated individuals who reported hearing about ZIKV first from their doctor/nurse compared to high school graduates (*p* = 0.03), and finally there were more high school graduates who correctly identified that ZIKV could be transmitted from mother to child (*p* = 0.03) than primary school educated individuals.

Based on the calculated knowledge score, the mean was 4.74, SD = 1.50, so all scores 1 standard deviation, (or higher than 6), were categorized as “adequate knowledge” and lower than 6 were classified as “low knowledge”. Using this classification, 72.2% (n = 117) were considered to have low knowledge and 25.9% (n = 42) were considered to have adequate knowledge.

### 3.3. Attitudes toward ZIKV

Nearly half of those who answered the perceived susceptibility HBM items (n = 77, 50%) agreed or strongly agreed that they were at risk for contracting the disease, yet less than 25% (n = 40) agreed or strongly agreed that they were at greater risk than others. The majority (n = 126, 77.8%) also agreed or strongly agreed that infection can cause severe consequences and the complications can be dangerous. The majority agreed or strongly agreed that preventing being bit by mosquitos (n = 108, 66.7%), using insect repellent (n =118, 72.8%), and wearing long pants/sleeves could prevent ZIKV transmission (n = 103, 63.6%), yet very few (n = 16, 9.8%) responded that using condoms would prevent the transmission of the disease (See Table 1).

### 3.4. Factors associated with Zika-related knowledge and attitudes

There was a statistically significant difference between gender and cues to action, (*p* = 0.04), with males having a statistically significant lower mean on the cues to action items (M = 10.51, SD ± 3.48) compared to females (M = 11.63, SD ± 3.51). Further analyses of the three cues to action items revealed a statistically significant difference between males and females on the item “My friends and family influence my decision to take action to prevent Zika Virus.” Male participants had a statistically significant lower mean (M = 3.25, SD ± 1.49) than females (M = 3.87, SD ± 1.43), indicating that women were more likely to agree that their family and friends influenced their decision to take action to prevent ZIKV.

There was a statistically significant difference on perceived severity between those categorized as having adequate knowledge compared to low knowledge on perceived severity (*p* = 0.03) (see Table 2). Further analysis showed that individuals with adequate levels of ZIKV knowledge endorsed more strongly the notion that “If I was to get ZIKV, it could spread to others in my family” (*p* = 0.03). There was also a difference in the perceived benefits construct (*p* = 0.04). Further analysis showed that individuals with higher levels of knowledge agreed more often with the item that “using insect repellent could prevent the spread of Zika (*p* = 0.00)”. There were no differences between educational level, rural/urban, or belief a vaccine exists for any of the HBM constructs.

**Table 4.** Summary of Zika Knowledge Item Frequencies by Gender, Location, and Educational Level (N = 181).

Item	Male (n = 85)	Female (n = 96)	*P*-Value	Rural (n = 87)	Urban (n = 94)	*P*-Value	Primary School (n = 41)	High School Graduate (n = 116)	*P*-Value
*Had heard of ZIKV*	76 (89.4)	86 (90.0)	1.00	78 (89.6)	84 (89.3)	0.95	37 (90.2)	102 (87.9)	0.69
*First heard about ZIKV:*									
Internet (Y)	17* (22)	8* (9.3)	**0.02**	12 (15.3)	13 (15.4)	0.99	8 (19.5)	16 (15.6)	0.42
Social media (Y)	18 (23.6)	26 (30.2)	0.35	20 (25.6)	24 (28.5)	0.68	10 (27.2)	26 (25.4)	86
Doctor/Nurse (Y)	2 (2.6)	8 (8.3)	0.11	8 (10.2)	2 (2.4)	0.05	5* (13.5)	3* (2.9)	**0.03**
Newspaper (Y)	3 (3.4)	1 (1.0)	0.25	3 (3.8)	1 (1.1)	0.35	2 (5.4)	2 (2.0)	0.29
TV (Y)	43 (57.0)	44 (51.1)	0.49	40 (51.2)	47 (56.0)	0.55	19 (51.3)	56 (54.9)	0.85
Radio (Y)	4 (5.0)	5 (5.8)	0.88	7 (8.9)	2 (2.3)	0.09	3 (8.1)	4 (3.9)	0.38
Friend (Y)	4 (5.0)	2 (2.3)	0.32	3 (3.8)	3 (3.5)	1.00	3 (7.3)	2 (2.0)	0.12
*ZIKV transmitted by:*									
Mosquitos (T)	71 (93.4)	80 (93.0)	0.92	72 (82.7)	79 (94.0)	0.66	35 (94.5)	94 (92.1)	0.62
Male Sexual Contact (T)	6 (7.0)	8 (8.3)	0.75	5 (5.7)	9 (9.6)	0.33	4 (9.8)	10 (8.6)	1.00
Mother to Child (T)	14 (16.4)	14 (14.6)	0.71	13 (14.9)	15 (16.0)	0.84	11* (26.8)	14* (12.1)	**0.03**
Blood transfusion (T)	12 (14.1)	17 (17.7)	0.51	13 (14.9)	16 (17.0)	0.69	3 (7.3)	21 (18.1)	0.57
Coughing/sneezing (F)	9 (10.6)	3 (3.1)	0.07	5 (5.7)	7 (7.5)	0.64	2 (4.9)	9 (7.8)	0.73
Vaccine (F)	38 (44.7)	43 (44.8)	1.00	35 (40.2)	46 (48.9)	0.21	20 (48.8)	50 (43.1)	0.70
Preventing mosquito bites (T)	49 (57.6)	53 (55.2)	0.71	46 (52.9)	56 (59.6)	0.31	22 (53.7)	68 (58.6)	0.43
Using condoms (T)	9 (10.6)	8 (8.3)	0.60	4* (4.6)	13* (13.8)	**0.04**	5 (12.2)	11 (9.5)	0.66
Preventing pregnancy (T)	5 (5.9)	12 (12.5)	0.13	7 (8.1)	10 (10.6)	0.54	7 (17.1)	8 (6.9)	0.06
*ZIKV transmitted by day biting mosquitos (Y)*	17 (20.0)	18 (18.75)	0.85	20 (23.0)	15 (16.0)	0.21	7 (17.1)	24 (20.7)	0.60
*Cases of ZIKV in Ecuador (Y)*	65 (76.5)	70 (72.9)	0.66	65 (74.7)	70 (74.5)	0.83	31 (75.6)	85 (73.3)	0.78
*Know someone with ZIKV (Y)*	12 (14.1)	11 (11.5)	0.65	16* (18.4)	7* (7.5)	**0.02**	7 (17.1)	12 (10.3)	0.30

Bolded * *p* < 0.05

## 4. Discussion

This pilot study was conducted to assess ZIKV-related knowledge and attitudes among adults in Ecuador using the framework of the HBM. At the time this study was conducted very few studies had assessed knowledge and attitudes of ZIKV. In this study (May 2016), nearly 90% of the Ecuadorian sample had heard of ZIKV, which is higher than in the Nigerian study [21] conducted in September 2016 (68.9%) and a study in the US in March 2016 (85%), but lower than in a follow-up nationally representative US sample (95.0%) conducted in September 2016 [21],[23]. This finding is not surprising since the current ZIKV epidemic started in Brazil and began impacting South and Central American countries before North America. To date, only one human case of ZIKV has been detected in Nigeria [21].

While overall knowledge level regarding transmission of ZIKV by mosquitoes was quite high (83.4%), there were many misconceptions related to ZIKV transmission and prevention. Less than 25% (n = 35) of the participants who had heard of ZIKV knew ZIKV was transmitted via day biting mosquitoes, which was not assessed in other studies. If participants assume that the virus is transmitted via night biting mosquitoes (like malaria), then they may not be likely to take precautions during the day, including using bug spray, or wearing protective clothing. ZIKV is transmitted by the same mosquitoes as dengue and chikungunya; but participants were not aware that these bite during the day.

Even fewer of the participants knew that ZIKV could be transmitted sexually, consistent with previous studies in other countries [20]–[23],[27]. Thus, few respondents understood that wearing condoms could prevent transmission. Participants may have believed they were protecting themselves from ZIKV via proper aforementioned preventive behaviors, but then acquired the virus through sexual activity. Condoms are available, but not highly used in Ecuador which is a predominantly Catholic country, even though the Pope condoned condom use to prevent ZIKV transmission [28]. Another barrier to condom use may be cost. Average condoms cost about $2.50 for a pack of 3; the average income for Ecuadorians is about $448 per month [29]. Female and male condoms are provided free of charge by the Ministry of Health in most of their public health centers and hospitals, however, participants need transportation and access to one of the health centers. Measures of behavior or intentions to perform prevention behaviors were not measured in this survey, only perceptions of self-efficacy for performing several prevention behaviors.

Finally, nearly half of the sample erroneously believed that a vaccine was able to prevent ZIKV. This may be because there was discussion early on about the creation of a vaccine on multiple media outlets in Ecuador, including the television and the Internet. We did not ask if they believed there was a vaccine that was developed and being withheld from them as some individuals in Africa and in the US believe has occurred with an HIV vaccine [30],[31] or if they erroneously believed that a vaccine was developed and available for prevention.

Few of the respondents knew someone who had been infected with ZIKV. This is not surprising since all but two of the locations (Amazon region) were regions where ZIKV was not endemic. Data were collected as a part of a routine trip to Ecuador and the locations were selected well in advance of this study. If this study had been done along the coastal region, or in Manabi and Esmeraldas Provinces where the large magnitude earthquake occurred in April 2016, the results may have been different. Furthermore, only a couple thousand cases of ZIKV have been confirmed in Ecuador. Many people may have been unknowingly infected with ZIKV, so it is not surprising that there are a limited number of Ecuadorians that would know one of the confirmed cases.

The data collection location and the low number of cases of ZIKV may also account for the low levels of perceived susceptibility; only about half of the participants strongly agreed or agreed that they were at risk for contracting the disease, and less than one third of the respondents strongly agreed or agreed that they were at greater risk than others. Regardless of the health issues, perceived susceptibly is almost always ranked lower than severity because of unrealistic optimism, which occurs when people rate their own circumstances or perceived risk for getting a disease or condition as lower than others people with the same level of risk [32]. This unrealistic optimism has been shown to occur in other studies assessing the HBM constructs [32]. The majority of participants agreed that the consequences of ZIKV are severe, but the likelihood that it will they will contract it is low. The “unrealistic optimism” may be a factor in this study since many of the participants lived in areas where ZIKV was not transmitted.

Furthermore, a strong perception of severity existed in this sample despite the fact that ZIKV infection is mild and for most, asymptomatic. ZIKV is severe for pregnant women and their babies, which were not the target of this study. Future research should explore the reasons behind the perceptions of severity. Emerging threats are perceived as severe when they have visible symptoms and have severe consequences, which is not the case for most people who are infected with ZIKV.

### Limitations

This study is not without limitations. First and foremost, recruitment is subject to selection bias. It is possible that those participants who agreed to participate were inherently different than those who chose not to participate. We also did not track the number of individuals who refused to participate in the study. While it was apparent that individuals in the rural, indigenous areas, were more likely to refuse to participate, it is not clear as to why they refused at a higher rate. It might be that they did not understand the purpose of the study as it was also clear that survey research was not something that most Ecuadorians had experience with or that they may have had lower levels of literacy and/or distrust. Also, the items developed for this study were only face validated and not assessed for reliability. It is possible that we were assessing the content in a manner that was well understood in the Ecuadorian population. Time did not allow for a pilot sample to be conducted, a lesson learned for future studies. There also was a small sample size. Again, this was a result of multiple factors, including limited time for data collection and a study instrument that was too lengthy. In future studies, the number of items collected will be limited to ensure that we are able to collect from more participants in the same amount of time. Refusal rates should be tracked in future studies to ensure meaningful reporting. Despite these limitations, this study adds to the ZIKV literature.

## 5. Conclusions

There is a clear need for education about the transmission and prevention of ZIKV across Ecuador to dispel myths and improve understanding. High levels of self-efficacy for prevention behaviors for ZIKV combined with low perceived barriers in this community set the stage for effective educational interventions or health promotion campaigns that can ameliorate the knowledge deficits surrounding transmission and prevention. It is forecasted that ZIKV will remain endemic in Ecuador; therefore, it is imperative that educational interventions coupled with increased access to and availability of condoms, bug spray, and protective clothing are needed to reduce the incidence of ZIKV as its associated consequences. In the future, more research is needed to better understand the factors contributing to ZIKV transmission in Ecuador so that interventions can be designed to be responsive to the needs of individuals living in parts of Ecuador where ZIKV is endemic.
